# Immune Reconstitution Kinetics following Intentionally Induced Mixed Chimerism by Nonmyeloablative Transplantation

**DOI:** 10.1371/journal.pone.0126318

**Published:** 2015-05-11

**Authors:** Nayoun Kim, Hyunji Lee, Junghoon Shin, Young-Sun Nam, Keon-Il Im, Jung-Yeon Lim, Eun-Sol Lee, Young-Nam Kang, Se-Ho Park, Seok-Goo Cho

**Affiliations:** 1 Laboratory of Immune Regulation, Convergent Research Consortium for Immunologic Disease, Seoul, Korea; 2 Institute for Translational Research and Molecular Imaging, The Catholic University of Korea College of Medicine, Seoul, Korea; 3 School of Life Sciences and Biotechnology, Korea University, Seoul, Korea; 4 Department of Radiation Oncology, Seoul St. Mary’s Hospital, The Catholic University of Korea College of Medicine, Seoul, Korea; 5 Catholic Blood and Marrow Transplantation Center, Seoul St. Mary’s Hospital, The Catholic University of Korea College of Medicine, Seoul, Korea; University of Lisbon, PORTUGAL

## Abstract

Establishing mixed chimerism is a promising approach for inducing donor-specific transplant tolerance. The establishment and maintenance of mixed chimerism may enable long-term engraftment of organ transplants while minimizing the use of immunosuppressants. Several protocols for inducing mixed chimerism have been reported; however, the exact mechanism underlying the development of immune tolerance remains to be elucidated. Therefore, understanding the kinetics of engraftment during early post-transplant period may provide insight into establishing long-term mixed chimerism and permanent transplant tolerance. In this study, we intentionally induced allogeneic mixed chimerism using a nonmyeloablative regimen by host natural killer (NK) cell depletion and T cell-depleted bone marrow (BM) grafts in a major histocompatibility complex (MHC)-mismatched murine model and analyzed the kinetics of donor (C57BL/6) and recipient (BALB/c) engraftment in the weeks following transplantation. Donor BM cells were well engrafted and stabilized without graft-versus-host disease (GVHD) as early as one week post-bone marrow transplantation (BMT). Donor-derived thymic T cells were reconstituted four weeks after BMT; however, the emergence of newly developed T cells was more obvious at the periphery as early as two weeks after BMT. Also, the emergence and changes in ratio of recipient- and donor-derived NKT cells and antigen presenting cells (APCs) including dendritic cells (DCs) and B cells were noted after BMT. Here, we report a longitudinal analysis of the development of donor- and recipient-originated hematopoietic cells in various lymphatic tissues of intentionally induced mixed chimerism mouse model during early post-transplant period. Through the understanding of immune reconstitution at early time points after nonmyeloablative BMT, we suggest guidelines on intentionally inducing durable mixed chimerism.

## Introduction

Solid organ transplantation has improved the survival in patients with end-stage organ diseases. However, the clinical success of organ transplantation has been mainly dependent on the use of long-term immunosuppressants. While advances in immunosuppression have reduced the incidence of acute rejection, these immunosuppressive drugs are generally nonspecific which often compromises graft function and causes various side effects, including infections and malignancies. Furthermore, current therapies still fail to prevent chronic rejection.

Allogeneic bone marrow stem cell transplantation (BMT) is a promising approach for the treatment of non-hematological diseases; however, high-dose chemoradiotherapy as a preparatory regimen for BMT is associated with incidence of acute graft-versus-host disease (GVHD) and substantial transplant-related toxicity. Despite the therapeutic potential of BMT, the associated risks have limited its broad application. For these reasons, many investigators have focused on developing conditioning strategies that enable the stable engraftment of allogeneic BM without causing severe immunosuppression in recipients [1,2]. Recent clinical and animal studies have shown that the use of nonmyeloablative conditioning regimens can overcome these obstacles [2–6]. The unique feature of nonmyeloablative conditioning is that it only partially destroys the recipient's hematopoietic system [1,7], allowing the coexistence of donor and recipient hematopoietic cells. In contrast to myeloablative conditioning which leads to full donor chimerism, nonmyeloablative conditioning preserves recipient immunity while minimizing risks of GVHD [8,9]. Therefore, the establishment of mixed chimerism by nonmyeloablative BMT is an attractive concept to induce long-term organ allograft tolerance.

Allogeneic mixed chimerism has been intentionally induced by various methods in murine models, and it is suspected that the immune reconstitution pattern immediately following BMT eventually allows for the development of mixed chimerism [4,9–11]. Therefore, understanding how infused donor BM cells and existing recipient cells are reconstituted after a nonmyeloablative regimen is important in achieving successful engraftment and mixed chimerism. However, the engraftment kinetics of specific hematopoietic lineages in mice with induced mixed chimerism have not been established, and only few studies have described the engraftment kinetics of full donor chimerism in humans [12–14]. In the present study, we outlined the kinetics of post-BMT expansion and the development of donor- and recipient-originated hematopoietic cells during the early post-transplant period. The aims of this study were to compare the recovery patterns for each hematopoietic cell lineage in various lymphatic tissues and to investigate the significance of these patterns of reconstitution. Through the extensive analysis of immune reconstitution following nonmyeloablative BMT, we suggest directions in the development of stable mixed chimerism.

## Materials and Methods

### Mice

Balb/c mice (H-2K^d^) were purchased from Charles River Japan, Inc. (Yokohama, Japan), while C57BL/6 mice (H-2K^b^) were purchased from Jackson Laboratories (Bar Harbor, MA). All mice were used at seven to ten weeks of age, unless otherwise specified. The mice were maintained under specific pathogen-free conditions in an animal facility with controlled humidity (55±5%), light (12/12h light/dark), and temperature (2261uC). The air in the facility was passed through a HEPA filtration system designed to exclude bacteria and viruses. Animals were fed mouse chow and tap water *ad libitum*. For blood collection, mice were anesthesized with 2.5% isoflurane in oxygen, and later sacrificed by CO_2_ exposure. Animal care and euthanasia protocols used in this study were approved by the Animal Care and Use Committees of Catholic University of Korea College of Medicine and Korea University (Permit number: 2010-0204-02).

### Preparation of mixed chimeras

Previously, we established a reliable method for inducing stable allogeneic mixed chimerism by host natural killer (NK) cell depletion and successful T cell-depleted donor BM grafts in fully MHC-mismatched nonmyeloablative BMT using a murine model [15]. Briefly, one day before BMT, recipient mice (eight-week-old Balb/c mice; n = 30) were injected intraperitoneally with 200 μl of phosphate-buffered saline (PBS) containing 40 μl of reconstituted anti-asialoganglioside GM1 antibodies with the concentration of 1mg/mL (40ug/mice) (Wako, Osaka, Japan). The recipient mice were then exposed to a single dose of 500 cGy of radiation from a Mevatron MXE-2 instrument (Siemens, New York, NY) with a focus-to-skin distance of 100 cm at a rate of 100 cGy/min. Donor BM cells were collected in Cedarlane cytotoxicity medium (RPMI 1640 medium supplemented with 25 mM HEPES buffer and 0.3% bovine serum albumin; Cedarlane, Hornby, Ontario, Canada) by flushing the shafts of the femurs and tibias of C57BL/6 mice.

Resuspended BM cells were depleted of T cells by incubation with anti-Thy-1.2 microbeads (Miltenyi Biotec, Auburn, CA) according to the manufacturer’s instructions. Within 6 h after irradiation, 2 x 10^7^ T cell-depleted BM cells in 0.5 ml of PBS were reinfused into the recipient mice.

### Flow cytometric analysis

The establishment of mixed chimerism in the spleen, mesenterial lymph nodes, bone marrow, and/or liver of recipient mice was monitored from week 1 to week 12 after BMT (n = 4 was included for each time point). Donor (H-2K^b^) and recipient (H-2K^d^) cells were distinguished during leukocyte gating by staining with allophycocyanin-labeled anti-H-2K^b^ (BD PharMingen) and PE-labeled anti-H-2D^d^ antibodies (BD PharMingen), respectively. To analyze the degree of mixed chimerism among the leukocytes, we performed surface staining using lineage-specific antibodies. The following conjugates were employed: FITC-tagged CD4 (clone RM4-5), TCRβ (clone H57), CD11c (clone HL3), and CD19 (clone 1D3) (all BD PharMingen); PE-tagged CD4 (clone RM4-5), TCRβ (clone H57), CD11c (clone HL3), CD45 (clone RA3-6B2), H-2D^d^ (cone 34-2-12), and mouse IgG1 (clone A85-1) (all BD PharMingen); peridinin chlorophyll-tagged Ly6G (Gr-1; clone RB6-8C5), CD8a (clone 53–6.7), TCRβ (clone H57), and CD45 (clone RA3-6B2) (all BD PharMingen); and allophycocyanin-tagged CD4 (clone RM4-5), TCRβ (clone H57), CD11c (clone HL3), CD45 (clone RA3-6B2), and K^b^D^b^ (clone R1.21.2) (all BD Pharmingen). NKT cells were detected by staining with CD1d dimer (DimerX, BD Biosciences) loaded with α-GalCer (KRN 7000, Funakoshi Co., Ltd) using the manufacturer's recommendations. After dimer staining, cells were stained with a fluorescent secondary antibody, allophycocyanin-tagged antimouse-IgG1 (clone X56, BD Pharmingen). The stained cells were then analyzed using a FACSCalibur with CELLQuest^TM^ software (both BD Biosciences). Only viable cells, as judged on the basis of their forward and side scatter, were used in the analyses. The percentage of donor-derived cells was calculated by dividing the percentage of donor cells by the total net percentage of donor plus recipient cells that showed positive staining for lineage-specific markers.

### Evaluation of GVHD

Survival was monitored daily after BMT, and the degree of GVHD was assessed weekly using a scoring system, which considered changes in five clinical parameters: weight loss, posture, activity, fur texture, and skin integrity. This scoring system is more accurate than previously described methods based on weight loss alone [16].

### Statistical analysis

The data are given as the mean ± SD. Comparisons between groups were made using Student’s *t*-test for independent samples; matched pairs were analyzed using Student’s paired *t*-test. Correlations were identified using Spearman’s rank correlation test.

## Results

### Lack of donor cell rejection following partial irradiation of the recipient

First, we depleted the NK cells from the recipients and T cells from the donor BM prior to BMT to prevent both host vs. graft rejection and graft vs. host rejection while inducing stable mixed chimerism. We assessed obvious signs of GVHD following post-BMT. The overall exterior, body weight, and feces of the recipient mice were monitored every day after BMT as markers of GVHD (data not shown). All mice used in this study survived until the time of analysis. No obvious abnormalities were detected and the body weights of the mice were consistent without post-BMT immunosuppressive treatment.

**Fig 1:**
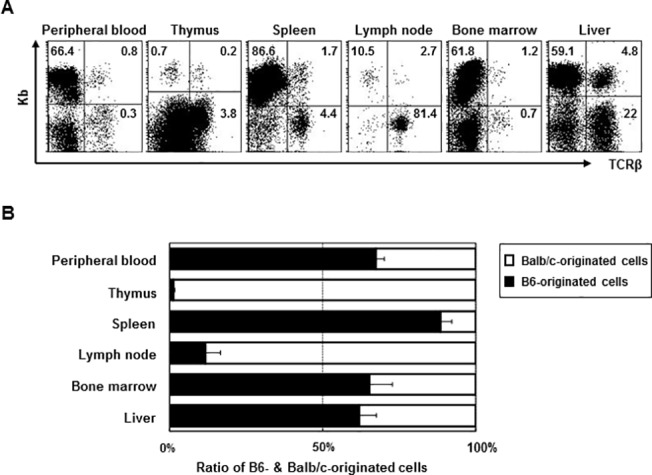
At one week after BMT, leukocytes isolated from the indicated organs were stained with anti-TCRβ,-K^b^, and-D^d^ monoclonal antibodies (*n* = 4). (A) The numbers in the dot plot show the percentage of B6-originated cells among the total leukocytes. (B) The bars show the ratio of B6- (■) and Balb/c-originated cells (□). Data are shown as the mean ± SD.

To further dissect the dynamics of the donor and recipient immune cells in the nonmyeloablative BM graft, we analyzed the leukocyte population in the tissues of the body between one and twelve weeks after BMT. Following *in vivo* anti-asialo GM1 antibody treatment, the majority of the host NK cells were successfully removed, and the T cells from the donor BM cells were also depleted by negative selection using MACS against anti-Thy1.2^+^ cells. The recipient mice, which were NK cell-depleted and partially irradiated, did not reject the transferred donor cells and allowed stable engraftment as early as one week after BMT (Fig 1). Since mature T cells in the donor BM were removed before BMT to reduce GVHD, donor-derived T cells in the recipients were not obvious until four weeks after BMT.

One week after BMT, we assessed the ratio of recipient to donor leukocytes originating in the peripheral blood, thymus, spleen, mesenterial lymph nodes (LN), BM, and liver. In all tissues except the thymus and lymph node, donor-derived cells constituted a large part of the cellular population, indicating that the transferred donor cells migrated to the target tissues and established residency without massive rejection by the host immune system, which received partial irradiation (Fig 1).

### Emergence of donor-derived T cells in the thymus and LN

**Fig 2:**
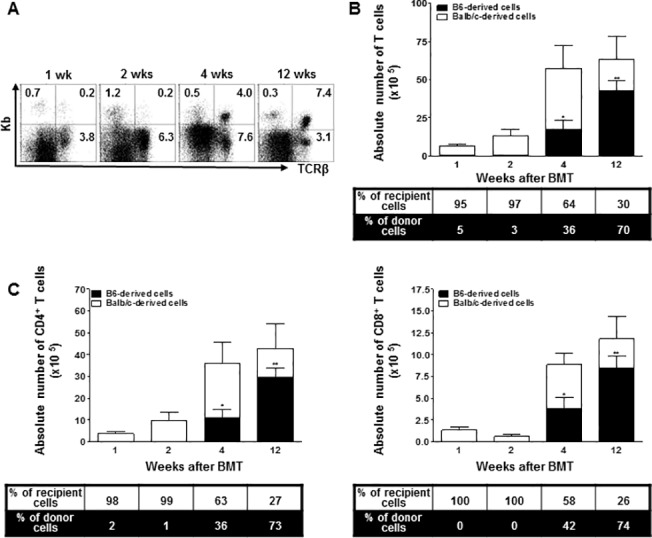
At weeks 1, 2, 4, and 12 after BMT, leukocytes isolated from the thymus were stained with anti-TCRβ,-CD4,-CD8,-K^b^, and-D^d^ monoclonal antibodies (*n* = 4 at each time point). (A) The numbers in the dot plot show the percentage of B6-originated T cells among the total leukocytes. (B) The bars show the ratio and absolute numbers of B6-orginated TCRβ^+^ T cells (■) and Balb/c-originated TCRβ^+^ T cells (□). (C) The bars show the ratio and absolute numbers of B6(Balb/c)-originated TCRβ^+^CD4^+^ and TCRβ^+^CD8^+^ T cells. Data are shown as the mean ± SD. p values compared to donor-derived cells one week after BMT. **, p ≤ 0.01, and *, p ≤ 0.05.

We monitored the T cells in the thymus and found C57BL/6-originated H-2k^b+^ T cells as early as one week after BMT (Fig 2A). The frequency of donor-derived T cells among the total leukocytes in the thymus did not change up to two weeks after BMT. By week 4, however, there was an increase in the absolute number of T cells, as well as the proportion of H-2Kb+ T cells. One third of the thymic T cells were of donor origin at this point, and the percentage continued to increase up to 60–70% at twelve weeks after BMT (Fig 2B). The CD4^+^ and CD8^+^ T cells showed a similar developmental pattern (Fig 2C).

**Fig 3:**
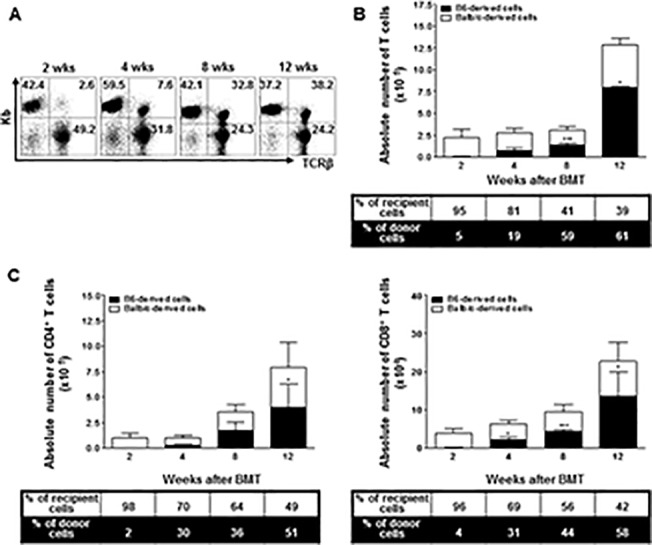
At weeks 2, 4, 8, and 12 after BMT, leukocytes isolated from mesenterial lymph nodes were stained with anti-TCRβ,-CD4,-CD8,-K^b^, and-D^d^ monoclonal antibodies (*n* = 4 at each time point). (A) The numbers in the dot plot show the percentage of B6-originated TCRβ^+^ T cells among the total leukocytes. (B) The bars show the ratio and absolute numbers of B6-orginated TCRβ^+^ T cells (■) and Balb/c-originated TCRβ^+^ T cells (□). (C) The bars show the ratio and absolute numbers of B6(Balb/c)-originated TCRβ^+^ CD4^+^ and TCRβ^+^ CD8^+^ T cells. Data are shown as the mean ± SD. p values compared to donor-derived cells two weeks after BMT. ***, p ≤ 0.0001 or p ≤ 0.001; **, p ≤ 0.01; and *, p ≤ 0.05.

We also monitored the ratio of donor-derived T cells in the mesenterial LN and found that the T cells followed the similar kinetics as those in the thymus. By week 2, donor-derived T cells comprised only a small proportion of the total lymph node cells, but their abundance increased to 30% by eight weeks after BMT. At the same time, the donor-derived cells among the total lymph node T cells increased up to 60% (Fig 3A and 3B). There was no noticeable difference between the CD4^+^ and CD8^+^ T cells in terms of their relative donor vs. recipient ratios (Fig 3C).

### Accumulation of T cells in the spleen and BM

**Fig 4:**
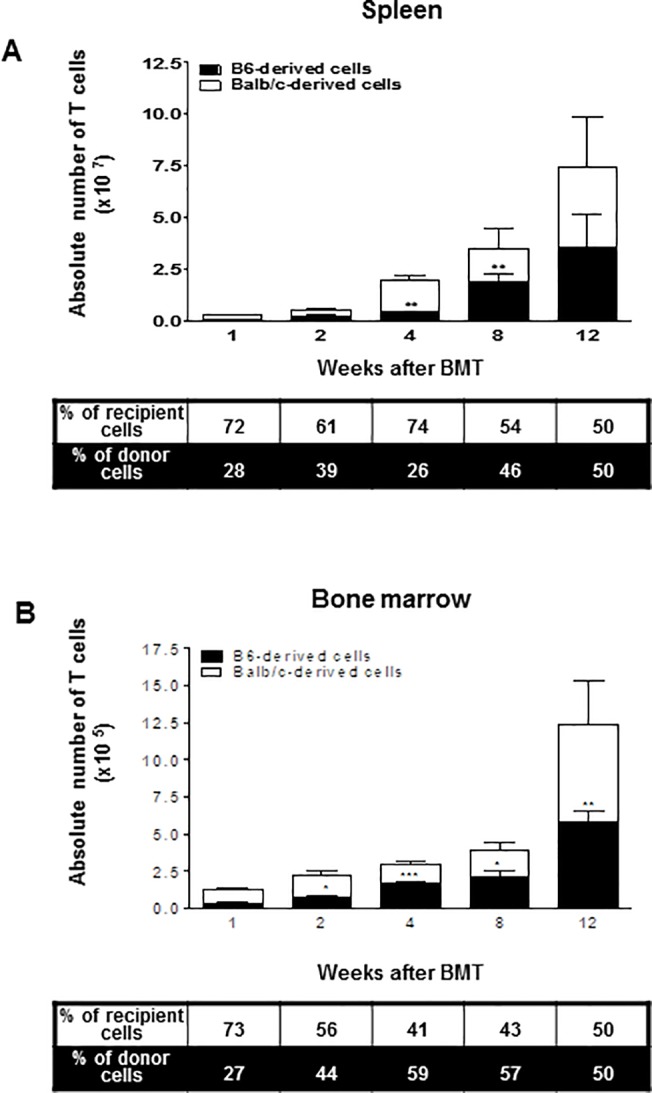
At weeks 1, 2, 4, 8, and 12 after BMT, leukocytes isolated from the spleen and bone marrow were stained with anti-TCRβ,-K^b^, and-D^d^ monoclonal antibodies (*n* = 4 at each time point). The bars show the ratio and absolute numbers of B6-orginated TCRβ^+^ T cells (■) and Balb/c-originated TCRβ^+^ T cells (□). Data are shown as the mean ± SD. p values compared to donor-derived cells one week after BMT. ***, p ≤ 0.0001 or p ≤ 0.001; **, p ≤ 0.01; and *, p ≤ 0.05.

In the spleen, one week after BMT, about 20% of the T cells were donor-derived, and beginning at two weeks after BMT, an exponential expansion in the absolute number of T cells was detected that lasted until week 12. By eight weeks after BMT, the donor and recipient T cells reached a ratio of about 1:1, which was maintained up to twelve weeks after BMT (Fig 4A). Furthermore, T cell recovery in the BM followed a pattern similar to that of splenic T cell development by twelve weeks after BMT (Fig 4B).

### NKT cell development

**Fig 5:**
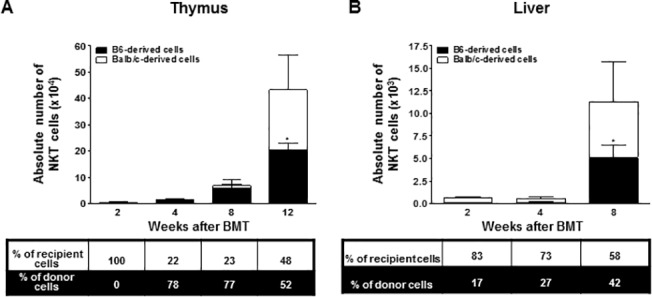
At weeks 2, 4, 8, and 12 after BMT, leukocytes isolated from the thymus (A) and liver (B) were stained with αGalCer-loaded CD1d dimer or anti-TCRβ,-K^b^, and-D^d^ monoclonal antibodies (*n* = 4 at each time point). The bars show the ratio and absolute numbers of B6-orginated NKT cells (■) and Balb/c-originated NKT cells (□). Data are shown as the mean ± SD. p values compared to donor-derived cells one week after BMT. *, p ≤ 0.05.

T cells expressing Vα14-Jα18 TCR were gated as typical CD1d-dependent NKT cells using CD1d/αGalCer-tetramer, and their origin was confirmed. At week 2 in the thymus, we were unable to detect NKT cells; however, they were detectable at weeks 4 and 8, although the population size was only modestly increased at this point. By twelve weeks after BMT, both the donor- and recipient-derived NKT cells had increased equally in number and become comparable to each other (Fig 5A). Next, we monitored the emergence of NKT cells in the liver, where NKT cells make up a prominent portion of the T cell population [17]. The number and ratio of donor-derived NKT cells were not obvious until four weeks after BMT, but increased to almost ten times the baseline number and encompassed 42% of all NKT cells by eight weeks after BMT (Fig 5B).

### Emergence of APCs

**Fig 6:**
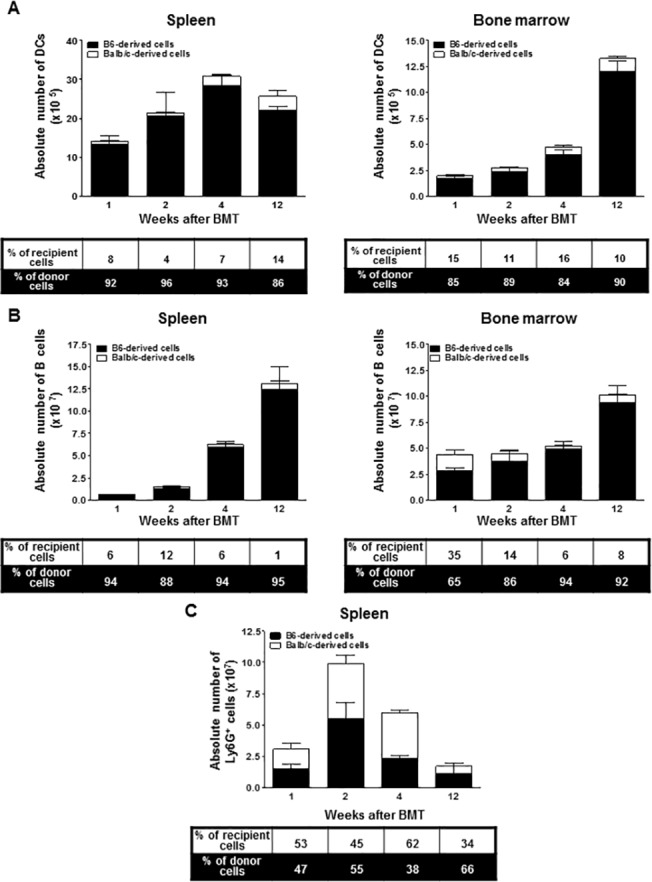
At weeks 1, 2, 4, and 12 after BMT, leukocytes isolated from the bone marrow and/or spleen were stained with anti-CD11c,-B220,-Ly6G,-K^b^, and-D^d^ monoclonal antibodies (*n* = 4 at each time point). (A) The bars show the ratio and absolute numbers of B6-orginated DCs (■) and Balb/c-originated DCs (□) in the bone marrow and spleen. (B) The bars show the ratio and absolute numbers of B6-orginated B cells (■) and Balb/c-originated B cells (□) in the bone marrow and spleen. (C) The bars show the ratio and absolute numbers of B6- (■) and Balb/c-originated Ly6G^+^ cells (□) in the spleen.). Data are shown as the mean ± SD.

The APCs, including DCs and B cells, in the recipients were mostly donor-derived cells early on after BMT (>90%). The DCs in the spleen and BM were largely donor-derived one week after BMT, and the total cell number increased modestly up to week 12 (Fig 6A). Most B cells were in the BM immediately after BMT whereas the number of B cells in the spleen was relatively low (Fig 6B). By four weeks after BMT, the number of splenic B cells had increased dramatically while the number of BM B cells was only slightly increased. By twelve weeks after BMT, both BM and splenic B cells were recovered at the level in normal mice, with a donor to recipient ratio of 9:1. Both recipient and donor-derived Ly6G^+^ cells were maintained at a 1:1 ratio immediately following BMT. Furthermore, there was an early increase in the number of Ly6G^+^ cells up to two weeks after BMT in the spleen indicating massive cell death and the clearance of transferred donor cells and irradiated recipient cells. At week 4, however, there was an overall reduction in the number of Ly6G^+^ cells, up to twelve weeks after BMT (Fig 6C).

## Discussion

The induction of mixed lymphohematopoietic chimerism may be promising in the field of solid organ transplantation because it can modulate the immunologic repertoire by extending the mechanisms of self-tolerance to donor-specific tolerance [18]. In the clinical setting, several studies have described transplant tolerance following allogeneic hematopoietic stem cell transplantation. Patients that received allogeneic transplantation could accept kidney [19–24], lung [25], liver [26] allografts from the original BM donor without immunosuppressive therapy. In these studies, however, patients developed organ failure as a complication of allogeneic BMT. Recently, efforts have been made to develop nonmyeloablative conditioning protocols to intentionally establish mixed chimerism for tolerance induction in patients receiving kidney [27–32] and liver [33–35] allografts. To achieve mixed chimerism, a delicate balance between donor and recipient immune cells is necessary. Therefore, in addition to specific conditioning regimens, the immune recovery pattern induced by such regimens may play a key role in the construction of durable mixed chimerism.

Donor T cells are known to play a significant role in promoting engraftment after allogeneic BMT, but can also cause GVHD, a major complication following allogeneic BMT [36]. Therefore, understanding the reconstitution kinetics of recipient- and donor-derived T cells is important in the clinical course following BMT. In our study, the population of donor-derived thymic T cells was minor two weeks after BMT (Fig 2), which represents newly developed T cells following the migration of T lymphocyte precursors introduced by BMT. These cells started to recover on a full scale at week 4 and reconstituted more than half of the donor (B6) cells by twelve weeks post-BMT. Donor-derived thymic CD4^+^ and CD8^+^ T cells showed similar recovery patterns in terms of their total numbers (Fig 2C). This was consistent with previous reports suggesting that CD4^+^ T cell recovery is relatively rapid and is similar to that of CD8^+^ T cells following nonmyeloablative regimens, while CD4^+^ T cells generally emerge later than CD8^+^ T cells in conventional conditioning[14,37]. Furthermore, CD4+CD8+ double-positive T cells are known to constitute a major population in the cortex during de novo T cell generation. Therefore, it will be important to discriminate between the single positive and double positive T cells in future studies to understand the thymus-dependent T cell reconstitution in mixed chimerism.

A similar pattern of T cell reconstitution was noted in the LN (Fig 3), which is a migration target for newly matured T cells. In contrast, donor-derived T cells in the spleen and BM already comprised a substantial portion of all T cells as early as one week after BMT (Fig 4). However, considering the extremely small number of T cells overall one and two weeks post-BMT, the significant portion of donor-type T cells in the spleen and BM might be the result of incomplete donor cell elimination during preparation. In the spleen, the total T cell number increased exponentially between weeks 2 and 4 post-BMT, and most of them were recipient-derived cells. These findings suggest that the recovery pattern of T cells differs depending on the organ examined. In addition, there are two sources for T cells in the recovering recipient: the peripheral expansion of mature T cells and the *de novo* production of naïve T cells in the recipient thymus derived from transplanted stem cells [38]. The exponential increase in the number of T cells between weeks 2 and 4 in the spleen might have resulted from the peripheral expansion of mature T cells; therefore, most of them were recipient-derived. However, in the thymus, LN, and BM, the increased number of T cells was largely dependent on the *de novo* production of naïve T cells from stem cells, and a significant portion of them were donor-derived.

In addition, NKT cells regulate immune responses and play an important role in the induction of allograft tolerance and suppression of acute GVHD [15,39]. However, they can also induce BM engraftment failure [40]. Therefore, understanding the delicate modulation of NKT cells is important. NKT cells are similar to T cells but are also characterized by such surface markers as NK1.1 in mice and CD161 in humans. Also, NKT cells develop later than conventional T cells [37,41]. We investigated whether the recovery pattern of NKT cells after nonmyeloablative BMT is similar in pattern to that of normal cells. We found that NKT cells developed in the thymus four weeks post-BMT (Fig 4A), and the donor- and recipient-derived NKT cells were increased equally in number twelve weeks after BMT. NKT cells did not appear in the liver until week 4 (Fig 4B), after which they rapidly increased in abundance up to eight weeks post-BMT. These results suggest that NKT cells recover later than conventional T cells, and they suggest differences in the developmental kinetics between the thymus and liver.

One remarkable finding of this study is the early increase in the number of donor-type APCs, including DCs and B cells, compared to other cell subsets (Fig 6). Although a significant portion of donor-derived APCs may have been transferred as part of the donor graft, the recovery was too rapid. Therefore, a considerable portion of the donor-derived APCs likely represent newly developed donor-derived cells. We postulate that APCs may suppress the development of T or NKT cells by secreting cytokines or other immunologic factors. Another possibility is that APCs delay the maturation of progenitor T or NKT cells through direct interactions. It has been previously reported that rapid donor APC generation may be associated with the development of donor-specific tolerance because APCs are capable of inducing the intrathymic deletion of alloreactive cells as well as mediating the establishment of peripheral tolerance [42,43]. In other words, APCs arising from a donor graft and persisting in a recipient are expected to present endogenous donor antigenic peptides, thereby inducing the deletion of alloreactive immature cells by negative selection. Consequently, other cell subsets that reconstitute later will not recognize donor-derived peptides as alloantigens. However, the precise mechanisms underlying the rapid recovery of APCs and their association with immune tolerance require further investigation. Furthermore, the kinetics of B cell development after BMT has not been fully studied in a murine model. In our study, we observed that the recovery of the total and donor-derived B cells was more prominent in the spleen than in the BM. It is natural for a significant portion of the transferred B cells to move to the spleen, which normally holds numerous naïve B cells. However, the mechanism that enables donor B cells to reside in the spleen at such an early time point is still unknown.

In summary, the exact mechanism underlying the development of immune tolerance in a mixed chimeric model has not been completely elucidated, but it is estimated that factors such as cytokines and reconstituted host- and donor-derived immune cells play an important role in inducing tolerance. In this regard, we have serially investigated the kinetics whereby immune cells of different hematopoietic lineages reconstitute and establish in various lymphatic tissues after a nonmyeloablative regimen in a murine model immediately after BMT, which is important for the stable engraftment of transplanted BM. Our results suggest guidelines for targeting the period of allograft susceptibility immediately after transplantation and inducing stable mixed chimerism. We believe that understanding the associated immune reconstitution kinetics may further improve the clinical feasibility of developing mixed chimerism for transplant tolerance.

## Supporting Information

S1 Checklist NC3Rs ARRIVE Guidelines Checklist. (PDF)
